# Multi-omics analysis reveals diagnostic and therapeutic biomarkers for aging phenotypes in ulcerative colitis

**DOI:** 10.1371/journal.pone.0338880

**Published:** 2025-12-17

**Authors:** Lei Guo, Jun Ge, Li Cheng, Xinyi Zhang, Zhengzheng Wu, Meili Liu, Hanmei Jiang, Wei Gong, Yi Liu

**Affiliations:** 1 Hubei Provincial Key Laboratory for Chinese Medicine Resources and Chinese Medicine Chemistry, School of Pharmacy, Hubei University of Chinese Medicine, Wuhan, China; 2 Hubei Shizhen Laboratory, Wuhan, China; 3 Pharmaceutical plant industry-university collaborative research center, Hubei University of Chinese Medicine, Wuhan, China; 4 Department of Oncology, Xiangyang Central Hospital, Hubei University of Arts and Science, Xiangyang, China; Versiti Blood Research Institute, UNITED STATES OF AMERICA

## Abstract

**Background:**

The incidence of ulcerative colitis (UC) remains high, with an increasing prevalence among elderly patients. Cellular senescence has been widely recognized as a contributor to UC susceptibility; however, the underlying molecular mechanisms remain incompletely understood. This study aimed to identify senescence-associated biomarkers in UC to provide new insight for diagnosis and treatment.

**Methods:**

By integrating transcriptomic data from UC patients with established aging-related databases, we identified aging-associated differentially expressed genes (DEGs). Using weighted gene co-expression network analysis (WGCNA) and Cytoscape, we pinpointed the core genes involved. A diagnostic model for UC was then developed based on these core genes, and their expression patterns were characterized at single-cell resolution. The roles of these genes were ultimately validated through in vitro and animal experiments.

**Results:**

We identified 24 aging-related DEGs in UC, which were primarily implicated in inflammatory responses and cytokine-receptor interactions. Further analyses pinpointed three core genes (CXCL1, MMP9, and STAT1) that were predominantly expressed in macrophages. A diagnostic model constructed using these genes exhibited robust predictive performance. Experimental validation confirmed that the expression levels of all three core genes were significantly upregulated in both a UC mouse model and in macrophages compared to controls. Additionally, pathway analyses revealed elevated levels of CXCL12 and VEGFA in the enriched pathways.

**Discussion:**

Our findings underscore the pivotal roles of CXCL1, MMP9, and STAT1 in UC-associated cellular senescence. The analysis positions these molecules as promising macrophage-mediated diagnostic biomarkers and therapeutic targets. Collectively, this work provides novel insights into UC pathogenesis and lays a foundation for developing precision medicine strategies that target senescence pathways.

## Introduction

Ulcerative colitis (UC) is a chronic and relapsing inflammatory bowel disease (IBD) characterized by persistent inflammation and epithelial damage of the colonic mucosa. Its clinical presentation is heterogeneous, primarily featuring persistent abdominal pain, diarrhea, and bloody stools with mucus. In severe cases, complications such as toxic megacolon and intestinal perforation may arise, imposing a substantial burden on patients’ quality of life [1,2]. Epidemiological studies indicate a rising global incidence of UC, a trend particularly pronounced among the elderly population [3,4]. Individuals over 60 years old account for approximately 20% of new diagnoses, with an incidence rate reaching 20–30 per 100,000 in this demographic [5]. Notably, both disease severity and the risk of complications escalate with advancing age [6]. This distinct age distribution suggests an intrinsic link between the aging process and UC pathogenesis.

Current diagnosis of UC relies on a combination of clinical manifestations, laboratory tests, and imaging studies, with colonoscopy and histopathological biopsy remaining the diagnostic gold standard [7]. However, the invasive nature and poor patient tolerance of endoscopy hinder its utility for routine follow-up monitoring. First-line therapeutic strategies include 5-aminosalicylic acid formulations, corticosteroids, immunosuppressants, biologics, and surgery. Although these treatments can ameliorate disease activity to some degree, they face considerable limitations. For instance, 20–40% of UC patients do not respond adequately to conventional medications [8]. Long-term use of glucocorticoids and immunosuppressants may also entail adverse effects, including increased infection risk, metabolic disturbances, and bone marrow suppression. Biologic therapies, while effective for some, are costly and associated with a risk of secondary loss of response. Therefore, a critical unmet need exists for the development of more precise, effective, and safer therapeutic strategies, as well as for non-invasive biomarkers to facilitate early diagnosis and long-term disease monitoring.

Recent research into the interplay between aging and chronic inflammatory diseases has established that the senescence-associated secretory phenotype (SASP) contributes to disease pathology by releasing a plethora of inflammatory factors and proteases, which amplify both local and systemic inflammation. This mechanism has been implicated in the progression of various conditions, including UC [9–11]. Supporting this, Levy et al.[12] demonstrated that aging is not merely an independent risk factor for UC but may actively drive its pathogenesis through mechanisms such as altered immune cell function, compromised intestinal barrier integrity, and dysregulated inflammatory signaling. Consequently, deciphering the mechanistic links between aging and UC is crucial for advancing diagnostic and therapeutic strategies for this disease.

This study implemented an integrated analytical strategy, combining bioinformatics mining of multiple databases with single-cell RNA sequencing (scRNA-seq) to systematically identify and validate core genes linked to aging phenotypes in UC. We delineated the expression patterns of these genes across diverse cell subtypes, thereby establishing a crucial link between UC and aging by integrating single-cell transcriptional profiles, immune microenvironment heterogeneity, and molecular regulatory networks. From the perspective of aging, our work identifies novel diagnostic biomarkers and therapeutic targets for UC. The expression of these targets in colonic tissue holds promise for developing rapid diagnostic tests, while our findings also provide a foundational insight for future drug development.

## Methods

### Gene collection and processing

Candidate genes were systematically screened through the analysis of public databases. UC microarray expression profiles were procured from the Gene Expression Omnibus (GEO) (https://www.ncbi.nlm.nih.gov/geo/), with GSE38713, GSE87473, and GSE179285 serving as the discovery cohort, and GSE47908, GSE48958, and GSE75214 comprising the validation cohort [13]. Detailed sample information for these datasets is provided in Supplementary S1 Table. A reference set of aging-related genes was constructed by integrating known aging-related genes from the CellAge (https://genomics.senescence.info/cells/) and GenAge (https://genomics.senescence.info/genes) databases [14,15]. This set was then supplemented with core genes involved in classical aging hallmark pathways, which were extracted from the Aging Atlas database (https://ngdc.cncb.ac.cn/aging) following the framework of López et al [16]. The combined gene lists were merged and deduplicated to form a consolidated aging-related gene set. Data processing followed a standardized workflow. The "limma" package was used for initial normalization and quality control of the GEO datasets. Where the necessary metadata were available, the lmFit function was subsequently employed to adjust for potential confounding factors, including sex, age, and batch effects. Following this, significantly differentially expressed genes (DEGs) were identified using thresholds of |log₂(fold change)| > 1 and an adjusted *p*-value < 0.05. The Benjamini-Hochberg (BH) method was applied consistently throughout this study to control the false discovery rate (FDR) for all multiple testing corrections, including the differential expression analysis.

### PPI network construction and analysis

The protein-protein interaction (PPI) network for the DEGs was constructed using the STRING database (version 11.5) with a confidence score threshold ≥ 0.4 [17]. The resulting network was imported and visualized in Cytoscape software. Hub genes were subsequently identified from this network by applying ten distinct ranking algorithms within the cytoHubba plugin [18]. The top candidates were visualized according to their degree of connectivity. Finally, these hub genes were submitted to the GeneMANIA database to predict their functional associations and to construct a refined protein interaction network [19].

### Consensus clustering and WGCNA

Given the significant disease heterogeneity of UC, we sought to define its clinical subtypes and molecular characteristics. To this end, we first integrated cohort data using the "sva" package, removing outlier samples based on Mahalanobis distance and Z-scores, and retaining genes with expression above the 25th percentile for mean and variance. This yielded a refined integrated dataset for downstream analysis. To balance the retention of informative features against the risk of high-dimensional noise, we calculated the expression standard deviation for each gene in the aging-related set across all UC samples. The top 200 most variable aging-related genes, ranked by descending standard deviation, were subsequently selected as features for consensus clustering. Unsupervised clustering was performed with the "ConsensusClusterPlus" package (parameters: maxK = 9, reps = 500, pItem = 0.8, pFeature = 0.8, K-means algorithm, Euclidean distance). We subsequently performed single-sample gene set enrichment analysis (ssGSEA) to quantify immune cell infiltration levels across the identified subgroups. Additionally, WGCNA was conducted to identify modules associated with clinical phenotypes [20]. A correlation matrix was constructed, and a soft threshold (R² ≥ 0.8) was applied to achieve a scale-free network. This matrix was converted into an adjacency matrix and then into a topological overlap matrix (TOM) to robustly identify co-expression modules by integrating both direct and indirect gene associations. The two modules most strongly correlated with the phenotypes were selected, and their intersection with the hub genes was determined.

### Gene enrichment analysis

To elucidate the biological pathways of the core genes, we performed KEGG pathway enrichment analysis in R. Gene symbols were converted to Entrez IDs using the org.Hs.e.g.,db package, and enrichment analysis was conducted with the enrichKEGG function from clusterProfiler. The top eight significantly enriched pathways (*p* < 0.05) are displayed. In parallel, Gene Set Enrichment Analysis (GSEA) was applied to the entire merged dataset to assess pathway-level changes. The GSEA utilized the "c2.cp.kegg.Hs.symbols" gene set and the GSEA function from clusterProfiler, with genes ranked by log₂(fold change) [21]. Pathways with an FDR < 0.05 were considered significant, and the top five based on normalized enrichment score (NES) are shown.

### Characterization of immune infiltration in UC

To assess the immune cell composition, we employed the CIBERSORT algorithm in R [22]. The normalized expression matrix was deconvoluted using the LM22 signature file with 1,000 permutations. Samples with a permutation *p*-value < 0.05 were retained to ensure reliability, and the resulting immune cell proportions were saved to "CIBERSORT-Results.txt". We then calculated a multi-gene combined score using the mean expression value of the core genes. This score was merged with the immune infiltration data from matched samples. Based on Spearman correlation analysis (*p* < 0.05), we generated a lollipop plot (ggpubr package) to display significant correlations and a separate scatter plot for key associations. Furthermore, the linkET package was used to create a comprehensive association plot, visualizing the relationships between core genes, immune cell infiltration levels, and the intercorrelations among different immune cell types.

### Single-cell RNA-seq analysis

We retrieved the single-cell RNA sequencing dataset GSE214695 from the GEO database using "ulcerative colitis" as the search term. The dataset comprised six healthy controls and six ulcerative colitis cases. Our analytical pipeline began with rigorous quality control measures employing the DoubletFinder package to predict and remove doublets while retaining only high-quality cells exhibiting mitochondrial gene content below 20% and expressing more than 200 genes. We further refined our analysis by focusing on genes demonstrating expression levels between 200–6000 counts and detected in at least three cells. The Seurat package served as our primary analytical framework for data integration. We performed log-normalization and identified the top 3000 variable genes using the FindVariableFeatures function. Dimensionality reduction was achieved using Uniform Manifold Approximation and Projection (UMAP) with the assistance of the singer R package, followed by comprehensive dataset integration and cell type annotation. We implemented six distinct algorithms (AUCell, UCell, singscore, ssgsea, JASMINE, and viper) in the irGSEA package to evaluate the expression patterns of core genes across cellular subpopulations. We used AUCell scoring to stratify macrophage and fibroblast subpopulations into high- and low-expression groups based on their mean expression thresholds. Pseudotemporal trajectory analysis was conducted using the Monocle package, where we constructed single-cell expression matrices to classify cells into distinct developmental states and delineate differentiation trajectories based on gene expression pattern recognition. The CellChat package, which has a default ligand-receptor interaction database (CellChatDB), was used for cell-cell communication analysis. This approach enabled us to identify cell type-specific interactions by detecting overexpressed ligands or receptors in particular cell populations. We also identified enhanced ligand-receptor interaction pairs associated with these overexpressed molecules. Complementing these analyses, we employed the R package Scenic to infer the activity states of the gene regulatory networks.

### Diagnostic model construction and validation

We performed logistic regression analysis on the three core genes and constructed a predictive nomogram using the "rms" package. To evaluate their diagnostic performance, we plotted receiver operating characteristic (ROC) curves for both individual genes and their combination in the training set using the "pROC" package. Subsequently, the multivariate model derived from logistic regression was applied to an independent validation set to confirm the diagnostic capability of the core genes. Additionally, Precision-Recall Curves (PRC) were generated to further assess the reliability of the diagnostic model. Beyond evaluating the predictive performance for distinguishing between the control and UC groups, we also assessed the ability of the three core genes to discriminate between molecular subtypes within the UC cohort, as defined by consensus clustering. Using the same clustering approach, we further validated the model’s classification performance within the UC samples of the validation set.

### Cell culture and experimental methods

The human colorectal adenocarcinoma Caco-2cell line was kindly provided by Professor Yi Liu, while the THP-1 monocytic cell line was obtained from the RSBM Public Cell Bank. Both cell lines were maintained at 37°C in a humidified 5% CO_2_ atmosphere using complete growth media supplemented with 10% fetal bovine serum (Batch No. G8002, Servicebio), 1% penicillin/streptomycin (Batch No. G4003, Servicebio), and either Dulbecco’s modified Eagle medium (Batch No. G4515, Servicebio) for Caco-2 cells or RPMI-1640 (Batch No. G4532, Servicebio) for THP-1 cells. For the experimental setup, each cell line was divided into control and model groups. An in vitro colitis model was established by treating Caco-2 cells in the model group with 10 μg/mL lipopolysaccharide (Catalog No. BS904, Servicebio) and collecting cell culture supernatants. THP-1 cells were differentiated into M0 macrophages through a 24-hour treatment with 100 ng/mL phorbol 12-myristate 13-acetate (Catalog No. P6741, Servicebio). Subsequently, control and model group M0 macrophages were cultured for 24 hours with the corresponding Caco-2 cell supernatants from either the control or model groups. Total RNA was extracted using an RNA isolation kit (Batch No. G3640, Servicebio) according to the manufacturer’s protocol. Primers for the CXCL1, MMP9, and STAT1 genes were purchased from Sangon Biotech Co., Ltd. (Shenzhen). PCR primers are shown in S2 Table. cDNA synthesis was performed using a standardized one-step RT-PCR kit (Batch No. G3329, Servicebio). Quantitative real-time PCR analysis was conducted using the SYBR Green SuperMix system (Batch No. G3326, Servicebio), and gene expression levels were calculated using the 2^-ΔΔCT^ method.

### Animal experimental methods

This study was approved by the Animal Experiment Ethics Committee of Xiangyang Central Hospital(Approval No., 2024-006-015). All animal experimental procedures were conducted in accordance with the “Guidelines for the Care and Use of Laboratory Animals in Xiangyang Central Hospital” and ARRIVE Guidelines 2.0. Five-week-old male specific pathogen-free BALB/c mice were procured from Hunan Silaikejingda Experimental Animal Co., Ltd. (Production License No. SCXK (Xiang) 2021−0002). All animals were housed under controlled environmental conditions maintained at 23 ± 3°C with 40 ± 10% relative humidity and a standardized 12-hour light/dark cycle. Following a 1-week acclimation period, the mice were randomly allocated to two groups (n = 15 per group): (1) the normal control group that received regular drinking water, and (2) the DSS model group administered 3% dextran sulfate sodium (DSS) in drinking water for 7 consecutive days to induce colitis. In addition, at the end of the experiment, we followed the American Veterinary Medical Association (AVMA) guidelines for euthanasia of animals (2020 edition). Euthanasia was performed by inhalation of 8% isoflurane anesthetic (INH, KW-MZJ-1, NJKEWBIO) for 6 minutes until the mice breathing and heartbeat completely stopped. And ensure that all operations are carried out by trained personnel to minimize animal suffering to the greatest extent possible.

### Histopathological and immunohistochemical analysis

A 1 cm segment of content-free colon tissue adjacent to the distal end was collected and fixed in 4% paraformaldehyde for 24 hours. Paraffin embedding and hematoxylin-eosin (HE) staining of colon sections were performed by Wuhan Servicebio Technology Co., Ltd. For immunohistochemical analysis, paraffin-embedded sections were sequentially processed through dewaxing and rehydration steps, followed by 3% methanol-hydrogen peroxide blocking. Antigen retrieval was performed with EDTA (pH 8.0) at 95°C for 30 minutes, followed by blocking with serum to inhibit non-specific binding. The sections were then incubated with primary antibodies at 4°C overnight, followed by 60-minute incubations with secondary antibodies and an ABC complex. Finally, diaminobenzidine (DAB) chromogenic substrate was applied for visualization. Tissue sections were probed with the following rabbit primary antibodies from Servicebio: anti-CXCL12 (GB11624, 1:1000) and anti-VEGFA (GB15165, 1:200). Antigen-antibody complexes were then detected using an HRP-conjugated goat anti-rabbit IgG secondary antibody (GB23302, Servicebio) at a 1:200 dilution.

### Western blot analysis

Protein extracts were prepared from tissues lysed in RIPA buffer supplemented with protease and phosphatase inhibitors (G2006, G2007, G2002; Servicebio). Following centrifugation at 12,000 rpm for 15 min at 4°C, the supernatant was collected, and protein concentration was determined using a BCA assay kit (ZJ101, Yamei). Proteins were separated by SDS-PAGE (10% or 15% gels) and transferred onto PVDF membranes. The membranes were blocked with 5% bovine serum albumin (GC305010, Servicebio) for 2 h at room temperature and then incubated overnight at 4°C with the following primary antibodies: Anti-CXCL12 (WL02283, Wanlei, 1:1000), Anti-MMP9 (WL03096, Wanlei, 1:1500), Anti-STAT1 (WL05088, Wanlei, 1:1000), anti-phospho-STAT1 (WL05288, Wanlei, 1:1500), anti-CXCL1 (12335–1-AP, Sanying, 1:1000), and anti-VEGFA (ET1604−28, Huaan, 1:1000). β-action (G15003, Servicebio, 1:5000) was used as a loading control. After washing with TBST, membranes were incubated with an HRP-conjugated goat anti-rabbit secondary antibody (GB23303, Servicebio, 1:10,000) for 2 h at room temperature. Protein bands were visualized using an ECL detection reagent. All experiments were performed in triplicate.

### Drug enrichment analysis and molecular docking

The DSigDB website (https://dsigdb.tanlab.org/DSigDBv1.0/) was accessed to download the "DSigDBv1.0_Detailed.txt" file [23]. The R packages "clusterProfiler", "org.Hs.e.g.,db" and "enrichplot" were used to perform enrichment analysis. A drug-gene interaction network was constructed based on adjusted *p*-values < 0.05. The three-dimensional structures of the target proteins and drug ligands were obtained from the PDB and PubChem databases, respectively. Proteins and ligands were preprocessed using PyMOL and AutoDock Tools, after which molecular docking was performed with AutoDock Vina. To evaluate the stability of the resulting complexes, molecular dynamics (MD) simulations were conducted using GROMACS. The protein was prepared using the CHARMM36 force field, and the system was solvated in a TIP3P water model and neutralized with ions (Na^+^/Cl^-^). Energy minimization was carried out using the steepest descent and conjugate gradient algorithms. The system was then equilibrated through 2 ns simulations under NVT and NPT ensembles, followed by a 100 ns production MD simulation.

### Data analysis

RT-qPCR, immunohistochemistry, and Western blot data were analyzed using GraphPad Prism software (version 10.1.2). Results are presented as mean ± standard deviation (SD). Comparisons between groups were performed using a grouped two-way ANOVA. A two-tailed *p*-value < 0.05 was considered statistically significant.

## Results

### PPI network and core nodes in UC mediated by aging-associated genes

**Fig 1:**
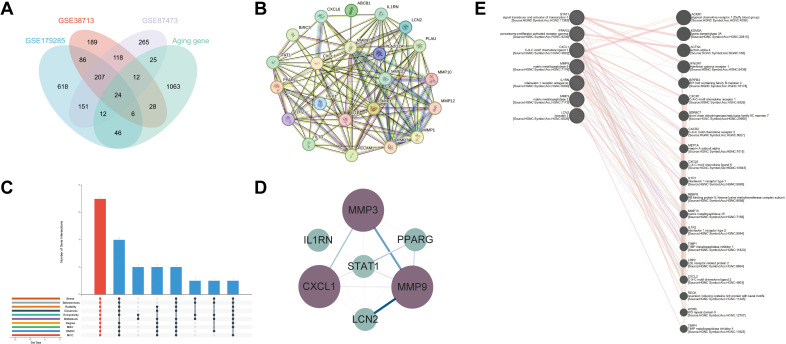
PPI Network. **A.** Intersection of the Senescence Gene Set and the UC Discovery Cohort; **B.** PPI Network of DEGs; **C.** Upset plot of DEGs; **D.** Visualization of hub genes using Degree values; **E.** PPI Network of Hub Genes.

Aging-related genes were sourced from the Aging Atlas, GenAge, and CellAge databases, containing 503, 307, and 949 genes, respectively. After merging and removing duplicates, a consolidated set of 1,217 aging-related genes was obtained. Characteristic gene were first identified in the UC datasets (GSE38713, GSE87473, GSE179285) using linear regression analysis. These Characteristic gene were then intersected with a predefined aging-related gene set, revealing a final set of 24 DEGs (Fig 1A). These 24 genes were imported into the STRING database to generate a PPI network (Fig 1B). Notably, the resulting network showed clustering around chemokine and metalloproteinase families, implying their potential bridging role between aging and UC pathogenesis. We then constructed an upset plot for the DEGs using 10 algorithms from CytoHubba and visualized the top 7 hub genes based on degree centrality (Figs 1C and 1D). Furthermore, the GeneMANIA database was used to predict functional associations among these hub genes under multiple network weighting models (Fig 1E). The analysis revealed 20 genes connected to the hub genes through physical interactions and co-expression networks. Most of these associated genes participate in biological processes including immune response, inflammation, cell migration, and tissue repair. This suggests that the core genes may function synergistically to coordinate key pathological processes—particularly inflammatory responses and tissue remodeling—thereby collectively contributing to disease progression. Additional details regarding co-expression modules (represented by different colors) and associations between network edges and disease phenotypes are provided in Supplementary S1 File.

### Integrative analysis of consensus clustering and WGCNA identifies aging-related gene modules in UC

**Fig 2:**
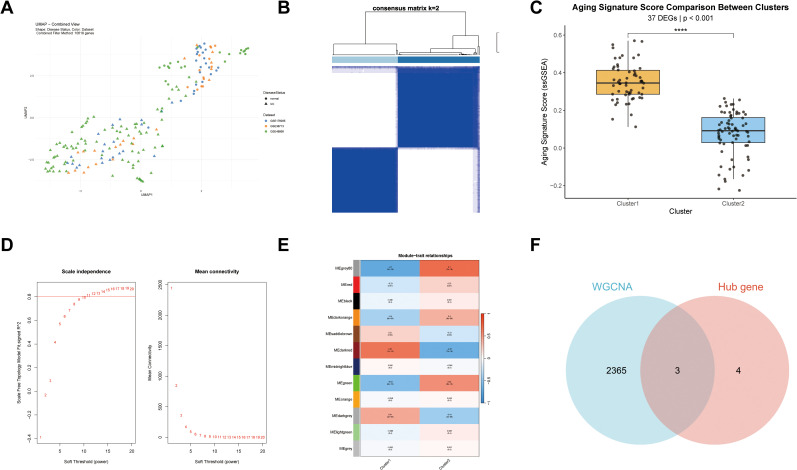
Molecular subtyping and core gene identification in UC. **A.** UMAP visualization of the training set cohort; **B.** Consensus clustering matrix illustrating sample relationships at k = 2; **C.** Box plots defining the two UC molecular subtypes based on aging-related gene expression; **D.** Scale independence and mean connectivity analysis for selecting the optimal soft-thresholding power in WGCNA; **E.** Module-trait relationships heatmap. Red and blue colors represent positive and negative correlations, respectively; **F.** Venn diagram identifying the core genes from the intersection of hub genes and key module genes. (WGCNA: darkred and darkgrey modules).

Following preprocessing, the merged dataset was normalized and subjected to principal component analysis (S1 Fig). This integrated dataset, comprising 57 normal controls and 130 UC samples, served as the training set and demonstrated excellent integrity, homogeneity, and biological plausibility in its sample distribution, confirming its suitability for downstream analyses (Fig 2A). Consensus clustering analysis of the 200 selected aging-related genes identified k = 2 as the optimal number, dividing the UC cohort into two distinct subgroups (Cluster 1, n = 58; Cluster 2, n = 72; Fig 2B). Subsequent analysis revealed that 37 of these 200 genes exhibited significantly elevated expression in Cluster 1 (*p* < 0.001; Fig 2C). Based on this enrichment of aging-related gene expression, we designated Cluster 1 as the “aging-associated UC subgroup” and Cluster 2 as the “non-aging subgroup”. Further comparative analysis of immune cell infiltration revealed that 20 immune cell types examined—including B cells, eosinophils, macrophages, and neutrophils—showed markedly distinct infiltration patterns between the two subgroups, revealing considerable immunophenotypic divergence within the UC cohort (S2 Fig). WGCNA employing a scale-free network model (soft threshold power = 13) identified 12 distinct gene modules (Figs 2D and 2E). Correlation analysis using module eigengene (ME) scores revealed that the darkred (r = 0.67, *p* < 0.001) and darkgrey (r = 0.41, *p* < 0.001) modules showed the strongest positive associations with the aging-associated UC subgroup. These modules contained 2,130 and 238 genes, respectively. Intersecting these module genes with previously identified hub genes yielded three core genes: CXCL1, MMP9, and STAT1 (Fig 2F). These core genes demonstrated significant differential expression not only between UC and control samples but also across the identified aging-related disease subtypes.

### Pathway enrichment profiling of whole-genome and core genes

**Fig 3:**
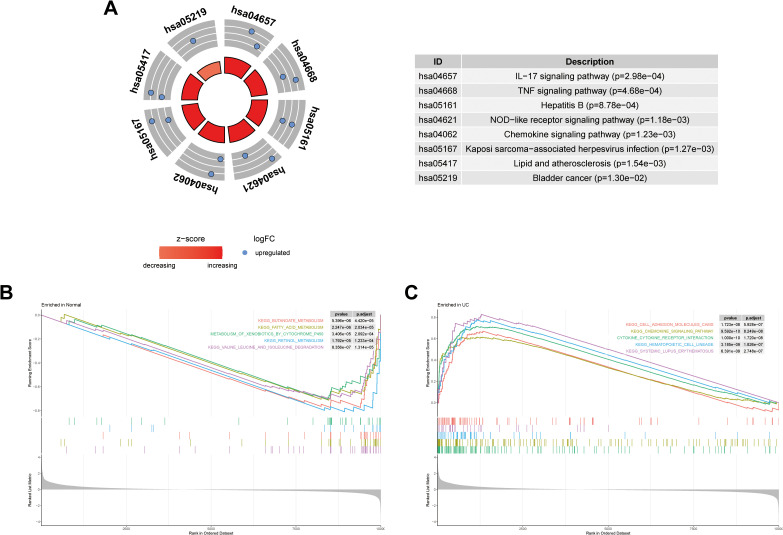
Pathway Enrichment Analysis. **A.** KEGG pathway enrichment analysis of the three core genes (CXCL1, MMP9, STAT1); **B.** GSEA plot for the normal control group; **C.** GSEA plot for the UC group.

KEGG pathway analysis of the three core genes identified significant enrichment in the IL-17 signaling pathway, TNF signaling pathway, and chemokine signaling pathway (*p* < 0.001; Fig 3A). Complementing this, Gene Set Enrichment Analysis (GSEA) of the entire training set transcriptome demonstrated that the chemokine signaling pathway and cytokine-cytokine receptor interactions were among the most highly enriched pathways in the UC group (*p* < 0.001; Figs 3B and 3C). The convergent evidence from both targeted and genome-wide analyses underscores the critical involvement of chemokine-mediated cytokine interactions in aging-related ulcerative colitis.

### Core genes shape the inflammatory immune landscape

**Fig 4:**
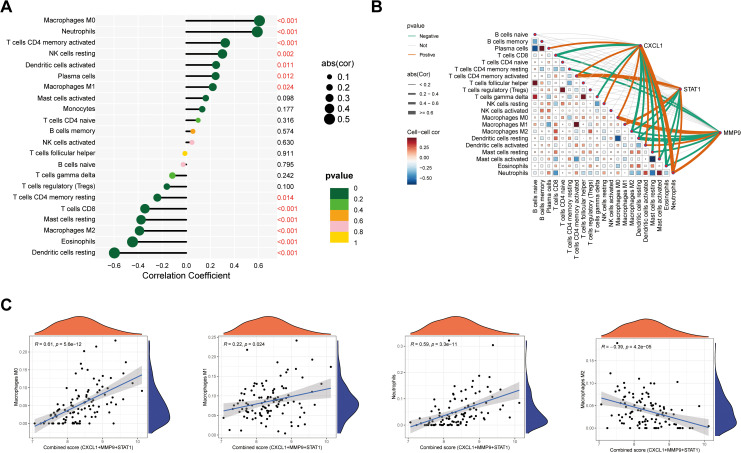
Immune infiltration analysis reveals core gene-immune cell associations. **A.** Lollipop plot showing the correlation between core gene expression and the infiltration levels of 22 immune cell types. Statistical significance is indicated (*p* < 0.05); **B.** Network diagram visualizing the correlation between core genes and immune cells (red edges: positive correlation; blue edges: negative correlation); **C.** Scatter plots illustrating the linear relationships between core gene expression and the abundance of significantly correlated immune cells.

Pathway enrichment analysis indicated that the core genes are involved in inflammatory and immune responses. Immune infiltration analysis further demonstrated that high or low expression of these core genes was significantly correlated with 13 of 22 immune cell types (*p* < 0.05). Key associations were observed with Macrophages M0, Neutrophils, T cells CD4 memory activated, and Macrophages M1 (Fig 4A). A correlation network revealed that the three core genes collectively promote the activation of Neutrophils, Macrophages M0, and Macrophages M1, while simultaneously suppressing Eosinophils, resting Dendritic cells, and Macrophages M2 (Fig 4B). Furthermore, significant intercellular immune interactions were identified, such as the positive correlation between T cells CD4 memory activated and Macrophages M1. Scatter plots visually confirmed the linear relationships between core gene expression and the abundance of significantly correlated immune cells (Fig 4C). These findings highlight the pivotal role of the core genes in reshaping the immune microenvironment, thereby elucidating a potential mechanism in UC pathogenesis.

### Expression and functional characterization of core genes

**Fig 5:**
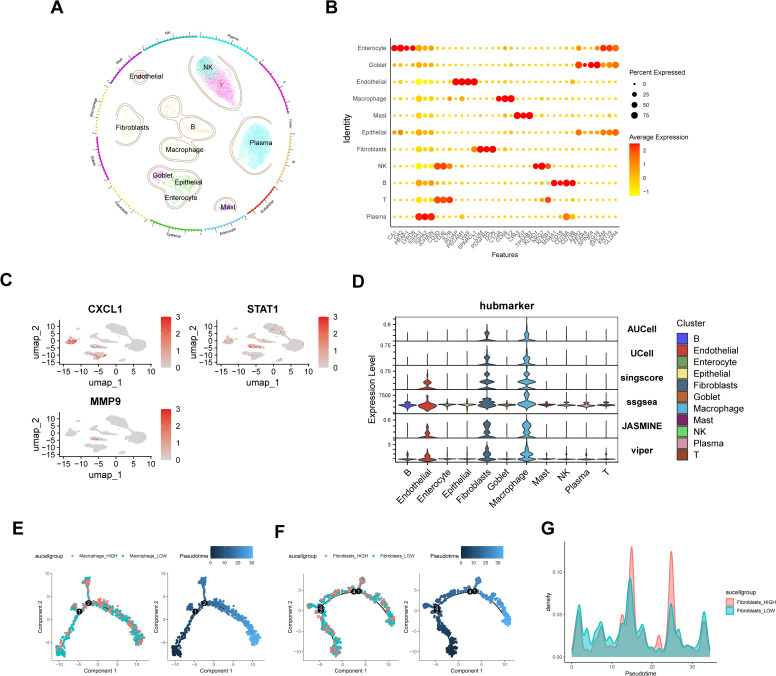
Cell Subpopulation Annotation and Trajectory Analysis. **A.** Cell Annotation Reveals 11 Distinct Cell Phenotypes; **B.** Bubble plot of relative expression of marker genes for each cell type; **C.** Distribution of three Hub Genes Across Cell Subpopulations; **D.** Expression Levels of Hub Genes Across Different Cell Types Using Six Computational Methods(AUCell、UCell、singscore、ssgsea、JASMINE and viper); **E.** Differentiation Trajectory and Pseudotemporal Distribution of Macrophages; **F.** Differentiation Trajectory and Pseudotemporal Distribution of Fibroblasts.

Following dataset preprocessing and Seurat pipeline analysis, the cells were clustered into 11 distinct types (Fig 5A), with cell types identified using canonical markers (Fig 5B). UMAP visualization demonstrated the predominant localization of three core genes within macrophage and fibroblast subpopulations (Fig 5C). This expression pattern was consistent with the results obtained from six computational algorithms (AUCell, UCell, singscore, ssgsea, JASMINE, and viper) (Fig 5D). Macrophage and fibroblast subsets were divided into two groups based on average AUcell scores. Pseudotemporal trajectory analysis was conducted using Monocle 2 and uncovered dynamic cellular behavior. Macrophage_HIGH cells exhibited progressive accumulation with concomitant reductions in differentiation potential throughout the developmental pseudotime (Fig 5E), while fibroblast_HIGH cells displayed expansion during medium differentiation phases before normalizing in later stages (Fig 5F).

**Fig 6:**
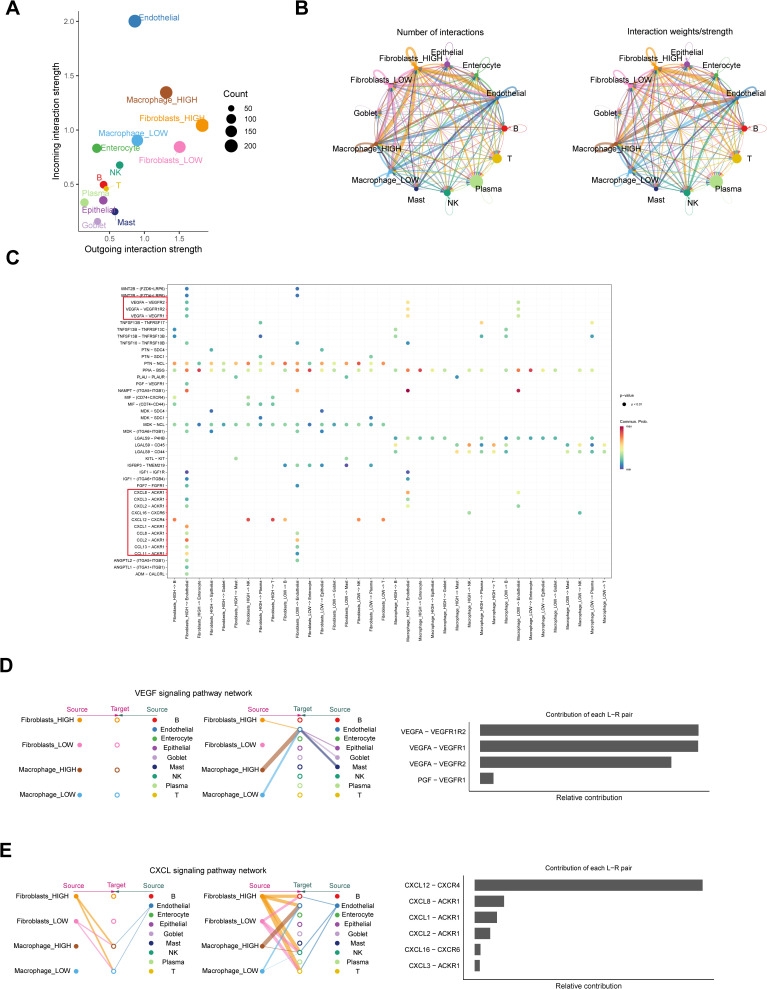
Cell Communication Analysis of Macrophage_HIGH and Fibroblasts_HIGH. **A.** Interaction Dynamics Between Different Cell Types; **B.** Intercellular Interaction Network Analysis Diagram; **C.** Bubble chart of macrophage and fibroblast interactions with various cell types via ligand-receptor interactions; **D.** Hierarchical interaction diagram of macrophage and fibroblast subpopulations with other cells in the VEGF signaling pathway; **E.** Hierarchical interaction diagram of macrophage and fibroblast subpopulations with other cells in the CXCL12 signaling pathway.

Cell communication analysis revealed significantly enhanced intercellular signaling activity in the macrophage_HIGH and fibroblast_HIGH groups compared to their LOW counterparts (Fig 6A). Although macrophages and fibroblasts interact with numerous cell types, macrophage_HIGH and fibroblast_HIGH subsets exhibit significantly stronger interactions with endothelial cells. This enhanced intercellular connectivity likely facilitates more robust signaling and material exchange (Fig 6B). Detailed ligand-receptor interaction mapping in UC mucosal tissues identified CXCL-(CXCR+ACKR) and VEGFA-(VEGFR1 + VEGFR2) as dominant signaling axes between macrophage_HIGH/fibroblast_HIGH and endothelial cells (Fig 6C). Systematic pathway network analysis was used to describe the critical role of macrophage_HIGH and fibroblast_HIGH cells in VEGF and CXCL signaling networks, and we found that fibroblasts and macrophages not only acted directly on endothelial cells, but macrophages were also stimulated by fibroblasts to varying degrees (Figs 6D and 6E).

### Validation confirms the robustness of the diagnostic model

**Fig 7:**
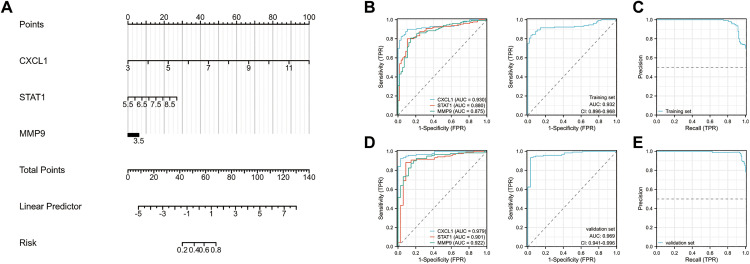
Disease Diagnostic Models. **A.** Diagnostic Nomogram Constructed from Three Hub Genes; **B.** ROC Curve of single gene in the Training Set; **C.** ROC and RP Curves for Multi-Gene Combinations in the Training Set; **D.** ROC Curve of single gene in the Validating Set; **E.** ROC and RP Curves for Multi-Gene Combinations in the Validating Set.

We constructed a diagnostic nomogram for UC based on the expression levels of CXCL1, MMP9, and STAT1 (Fig 7A). All core genes showed a positive correlation with disease occurrence. ROC analysis was performed on both single-gene and multi-gene combination models in the training set, with an area under the curve (AUC) > 0.7 considered indicative of strong diagnostic value. The multi-gene model achieved a significantly superior AUC of 0.932 compared to individual genes, demonstrating excellent classification performance within the training set (Figs 7B and 7C). Applying the same nomogram-derived risk scores to the independent validation set, the combined model maintained an outstanding AUC of 0.969, confirming its robust diagnostic reliability (Figs 7D and 7E). Furthermore, the three-gene signature also exhibited remarkable predictive capability for distinguishing molecular subtypes within the UC cohort, with combined AUC values exceeding 0.9 in both training and validation sets. These significant results indicate that our diagnostic biomarkers not only effectively identify UC, but more importantly, successfully discriminate between distinct aging-related molecular subtypes within this disease, highlighting their potential clinical utility for patient stratification (S3 Fig)

### Upregulation of core genes in the UC model

**Fig 8:**
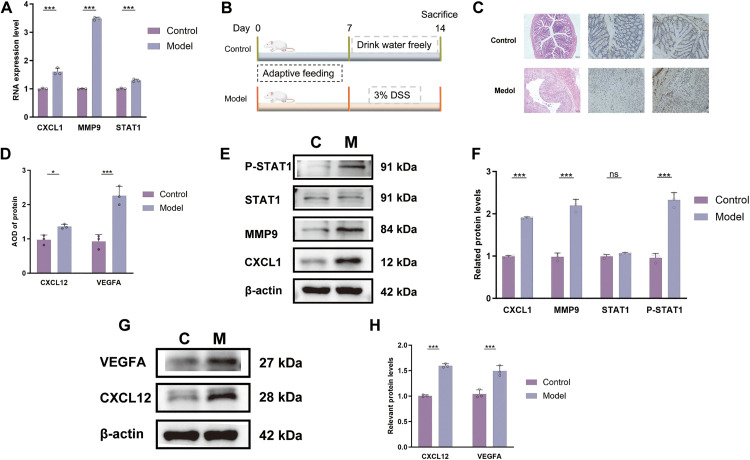
Experimental validation of core gene expression and associated pathological changes. **A.** mRNA expression levels of the core genes (CXCL1, MMP9, STAT1) in colon tissues; **B.** Schematic flowchart of the animal experimental design; **C.** Representative H&E staining and immunohistochemistry (IHC) images of colon tissue sections; **D.** Protein expression levels of VEGFA and CXCL12 as quantified from IHC; **E.** Immunoblotting results for the core gene-encoded proteins; **F.** Quantitative analysis of core gene protein expression from immunoblotting; **G.** Immunoblotting results for CXCL12 and VEGFA proteins; **H.** Quantitative analysis of CXCL12 and VEGFA protein expression from immunoblotting (*P < 0.05, **P < 0.01, ***P < 0.001).

Quantitative real-time PCR analysis revealed significantly elevated expression levels of CXCL1 (*p* < 0.001), MMP9 (*p* < 0.001) and STAT1 (*p* < 0.001) in the model group compared to the controls (Fig 8A). These findings are consistent with our data analysis, providing experimental validation for the pivotal role of these core genes in the pathogenesis of UC.BALB/c mice were induced by DSS to establish UC models (Fig 8B). Histopathological examination revealed distinct morphological differences between the groups. Control mice exhibited intact colonic epithelium with well-organized goblet cell distribution and preserved crypt architecture. In contrast, DSS-treated mice showed extensive mucosal damage characterized by inflammatory cell infiltration, ulceration, and severe crypt distortion (Fig 8C). Immunohistochemical analysis further confirmed the markedly elevated expression of CXCL12 (*p* < 0.05) and VEGFA (*p* < 0.001) in model group specimens compared to controls (Figs 8C and 8D). In addition, the results of Western blotting also showed that the expression levels of core genes CXCL1 (*p* < 0.001), MMP9 (*p* < 0.001) and p-STAT1 (*p* < 0.001) and pathway expression genes CXCL12 (*p* < 0.001) and VEGFA (*p* < 0.001) in the model group were significantly higher than those in the control group (Figs 8E–8H).

### Computational discovery of therapeutic candidates for UC

**Fig 9:**
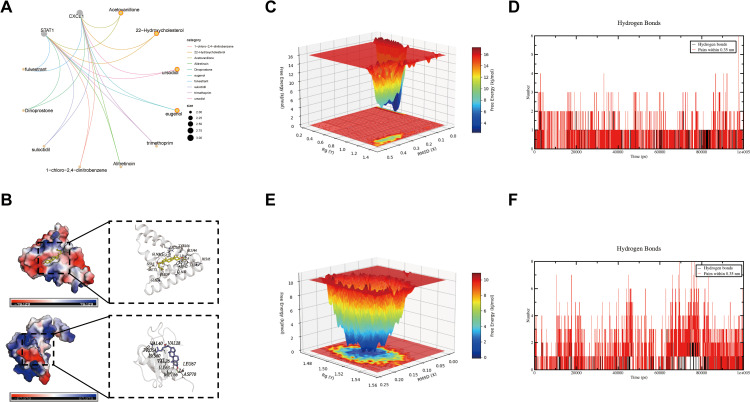
Computational drug screening and molecular docking analysis. **A.** Interaction network between hub genes and potential therapeutic compounds. Node size corresponds to the number of targeted genes; **B.** Molecular docking poses of 22-hydroxycholesterol with CXCL1 (top) and STAT1 (bottom); **C.** Three-dimensional free energy surface for the CXCL1/22-hydroxycholesterol complex; **D.** Detailed view of hydrogen bonding interactions in the STAT1/22-hydroxycholesterol complex; **E.** Three-dimensional free energy surface for the STAT1/22-hydroxycholesterol complex; **F.** Secondary confirmation of hydrogen bonding patterns in the STAT1/22-hydroxycholesterol complex.

We screened the DSigDB database to construct a drug-gene interaction network, selecting significantly enriched candidates (*p* < 0.05) for visualization (Fig 9A). In the resulting network, the node size for each drug corresponds to the number of core genes it targets, with larger nodes indicating broader potential pharmacological scope. Molecular docking energies between the candidate drugs and core genes are summarized in Table 1. We further visualized the most stable docking poses (Figs 9B–9F) and validated binding stability through molecular dynamics simulations. Our integrated computational approach identifies 22-Hydroxycholesterol as a particularly promising therapeutic candidate for ulcerative colitis.

**Table 1:** Molecular docking results between core genes and candidate drugs.

Receptor	Ligand	CAS	Affinity (kcal/mol)
**CXCL1**	Acetovanillone	498-02-2	−4.3
**P09341**	22-Hydroxycholesterol	17711-16-9	−6.4
	ursodiol	128-13-2	−6.2
	eugenol	97-53-0	−4.2
**STAT1**	Acetovanillone	498-02-2	−4.8
**P42224**	22-Hydroxycholesterol	17711-16-9	−6.8
	ursodiol	128-13-2	−6.4
	eugenol	97-53-0	−4.8

## Discussion

Accumulating studies have shown that immune dysfunction with aging is closely related to chronic inflammation [24]. He et al.[25] and Zhou et al.[26] also reported that this effect is particularly prominent in UC. The available evidence suggests that aging not only leads to dysbiosis of the microflora but also that the senescence of intestinal stem cells limits intestinal mucosal repair and accelerates UC progression [27,28]. In addition, it is noteworthy that the colon characteristics of senescent animals have similarities with UC [29], once again validating the important role of senescence in the development of UC. Although these studies have made some progress in understanding senescence and UC, the molecular model of how senescence drives the development of UC has still not been adequately demonstrated, hampering the generation of new therapeutic modalities for UC.

To elucidate the pivotal role of aging-related genes in the pathogenesis of UC, this study identified 24 aging-associated genes linked to UC through integrative analysis of multiple UC transcriptomic datasets and established aging-related gene sets. Given the challenge of disease heterogeneity in UC, we further employed consensus clustering and WGCNA to screen for aging-related genes that robustly distinguish UC samples. This approach led to the identification of three core aging genes—CXCL1, MMP9, and STAT1—that play central functional roles in UC progression. CXCL1, a classical inflammatory chemokine, modulates inflammatory responses and vascular remodeling [30]. MMP9 is involved in extracellular matrix degradation and cytokine regulation, while STAT1, a key signaling molecule, participates in the regulation of apoptosis, proliferation, and the cell cycle [31]. Immune infiltration and functional enrichment analyses revealed that high expression of these core genes actively contributes to inflammatory processes and tissue remodeling in the colonic mucosa during UC progression. Previous studies have corroborated these findings, demonstrating that inhibition of CXCL1 or STAT1 in murine models of ulcerative colitis alleviates colonic inflammation. Additionally, serum MMP9 levels are significantly elevated in UC patients compared to healthy controls [32–34]. Moreover, a diagnostic model constructed using these core genes exhibited a combined predictive accuracy exceeding 0.9, demonstrating robust performance. Collectively, these results substantiate the critical involvement of core aging-related genes in UC pathogenesis and underscore their potential as promising therapeutic targets for disease management.

The critical role of core genes in UC development is well recognized; however, the specific mechanisms through which these genes contribute to UC pathogenesis remain incompletely understood. To address this, we performed single-cell analysis to delineate the functional patterns of core genes in UC. We comprehensively identified 11 distinct cell types based on signature marker genes and localized the expression of core genes primarily to macrophage and fibroblast subpopulations [35]. Cell communication analysis revealed that macrophage_HIGH and fibroblast_HIGH subsets—which exhibit high expression of core genes—show significantly enhanced interactions with endothelial cells compared to other cell types. Further investigation of receptor–ligand interactions indicated that Macrophage_HIGH directly mediates VEGF signaling pathways in endothelial cells. Within the CXCL signaling pathway, both Macrophage_HIGH and Fibroblast_HIGH not only directly activate endothelial cells but also facilitate the activation of stromal immune cells by fibroblasts, thereby amplifying inflammatory and remodeling processes. These findings are consistent with trajectory analysis, which demonstrated that macrophage_HIGH reaches peak activity during disease progression, whereas fibroblast_HIGH increases initially but returns to baseline as the disease advances. To validate these observations, we established cellular and animal models that closely mimic human UC pathophysiology [36]. In vitro experiments showed significantly elevated expression of core genes in macrophages, and in vivo assessments using immunohistochemistry and Western blotting further confirmed the critical involvement of VEGF and CXCL signaling pathways. Previous studies have reported that intestinal secretion of VEGFA and CXCL chemokines exacerbates intestinal inflammation and disrupts epithelial barrier integrity [37,38]. In light of our data, we propose that the three core aging genes accelerate UC progression by mediating inflammatory infiltration and vascular remodeling, primarily through macrophage-driven activation of VEGF and CXCL signaling in endothelial cells. Additionally, through computational drug screening, we identified 22-hydroxycholesterol as a candidate therapeutic agent. Although still in the exploratory stage and not yet supported by clinical evidence or guideline recommendations, 22-hydroxycholesterol has been shown to inhibit the NF-κB pathway and prevent its nuclear translocation, thereby suppressing the transcription and release of pivotal pro-inflammatory cytokines—such as TNF-α, IL-6, and IL-1β—which are central to UC pathogenesis [39]. In summary, the three core aging genes show promise as diagnostic biomarkers. Based on their mechanistic roles, targeting macrophages with immunosuppressants or inhibitors of these core genes may represent a valuable therapeutic strategy for UC. However, several limitations of this study should be acknowledged. First, although our animal studies confirm the importance of the core genes in UC pathogenesis. However, to definitively establish their specificity as biomarkers for the 'aging-related UC subtype,' future studies should include functional experiments using aged animal models or systems that better mimic human disease heterogeneity, such as patient-derived organoids. Second, while our integrated computational approach identified promising drug candidates-particularly 22-Hydroxycholesterol-their actual therapeutic efficacy and safety require experimental validation in suitable preclinical models. Finally, although cell-cell communication analysis revealed functional interplay between fibroblast and macrophage subpopulations, the precise molecular mechanisms underlying this crosstalk remain to be fully elucidated. Future work incorporating co-culture systems and spatial transcriptomics will help clarify how fibroblasts modulate macrophage activation during UC progression..

## Conclusion

The present study combined multi-omics analysis with experimental validation to identify three senescence genes associated with UC (CXCL1, MMP9 and STAT1) and preliminarily verified that the core genes play a key role in UC through macrophage/endothelial-mediated mechanisms. The mechanism by which fibroblasts activate macrophages to promote UC development is proposed. These results are expected to provide new targets for the diagnosis and treatment of the UC senescence phenotype.

## Supporting information

S1 Fig Data preprocessing and quality control analysis. A. Baseline characteristics of the training set prior to standardization; B. Baseline characteristics of the training set following standardization; C. Principal component analysis (PCA) of the training set colored by disease status; D. PCA of the training set colored by dataset source; E. Baseline characteristics of the validation set prior to standardization; F. Baseline characteristics of the validation set following standardization; G. PCA of the validation set before batch effect correction; H. PCA of the validation set after batch effect correction.
(TIF)

S2 Fig Immune infiltration analysis of Cluster1 and Cluster2. (*P < 0.05, **P < 0.01, ***P < 0.001).
(TIF)

S3 Fig Intra-UC Group Diagnostic Models. A. Intra-UC group diagnostic scatter plot constructed from the expression levels of three core genes; B. ROC curve for single genes within the UC group in the training set; C. ROC curve for multi-gene combinations in the training set; D. ROC curve for single genes within the UC group in the validation set; E. ROC curve for multi-gene combinations in the validation set.
(TIF)

S1 Table Sample information for the ulcerative colitis transcriptome dataset. (DOCX)

S2 Table Primer sequences for quantitative PCR analysis of core genes. (DOCX)

S1 File Protein interaction network of hub genes. (PDF)

S1 Data Raw data. (ZIP)
