# Supplementary figures and images for: Multi-omics analysis reveals diagnostic and therapeutic biomarkers for aging phenotypes in ulcerative colitis
Source: PLoS One. 2025 Dec 17;20(12):e0338880. doi: 10.1371/journal.pone.0338880 (PMC12711006; doi:10.1371/journal.pone.0338880)

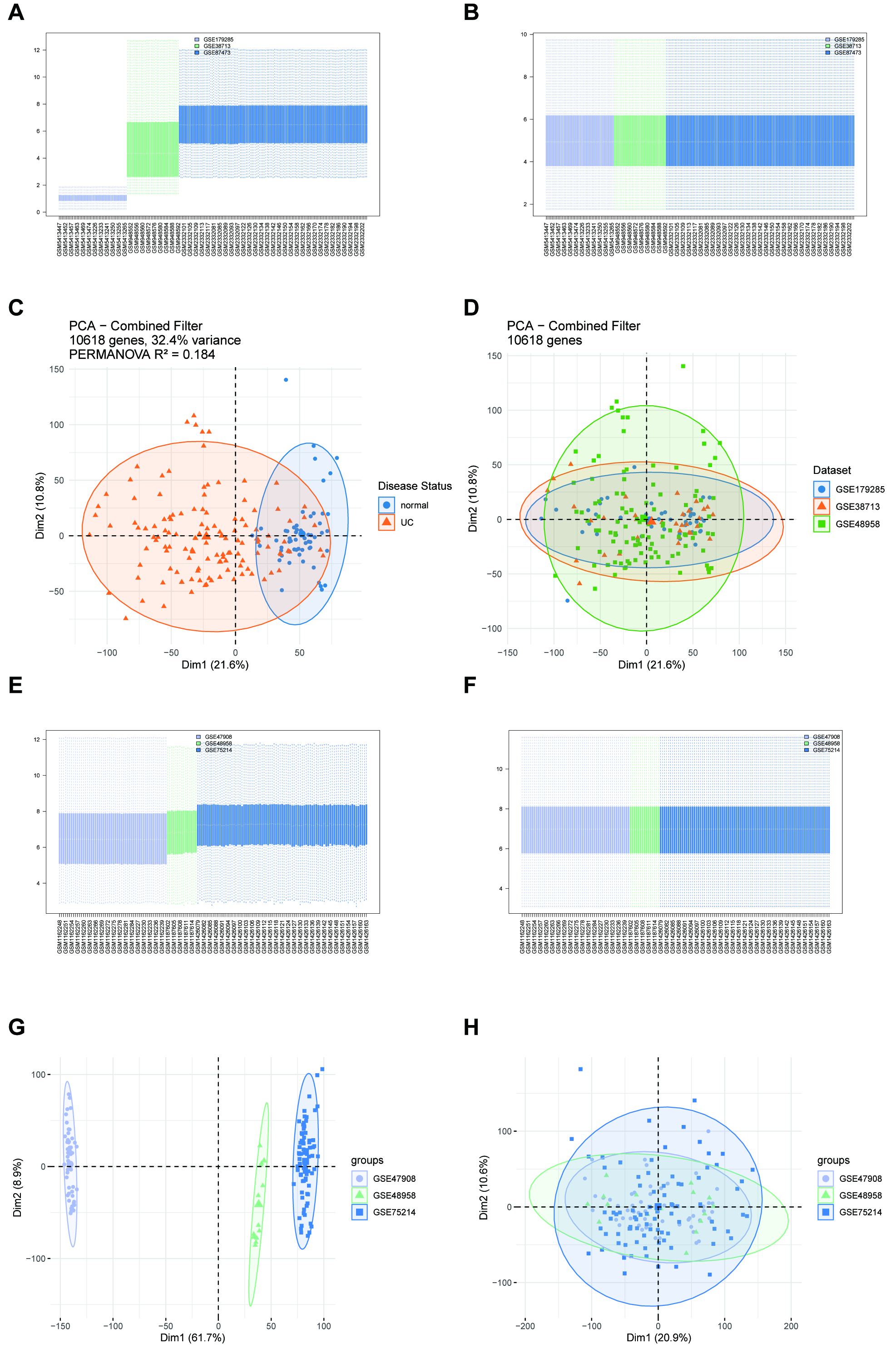

Supplement: S1 Fig — A. Baseline characteristics of the training set prior to standardization; B. Baseline characteristics of the training set following standardization; C. Principal component analysis (PCA) of the training set colored by disease status; D. PCA of the training set colored by dataset source; E. Baseline characteristics of the validation set prior to standardization; F. Baseline characteristics of the validation set following standardization; G. PCA of the validation set before batch effect correction; H. PCA of the validation set after batch effect correction. (TIF) [file pone.0338880.s001.tif]

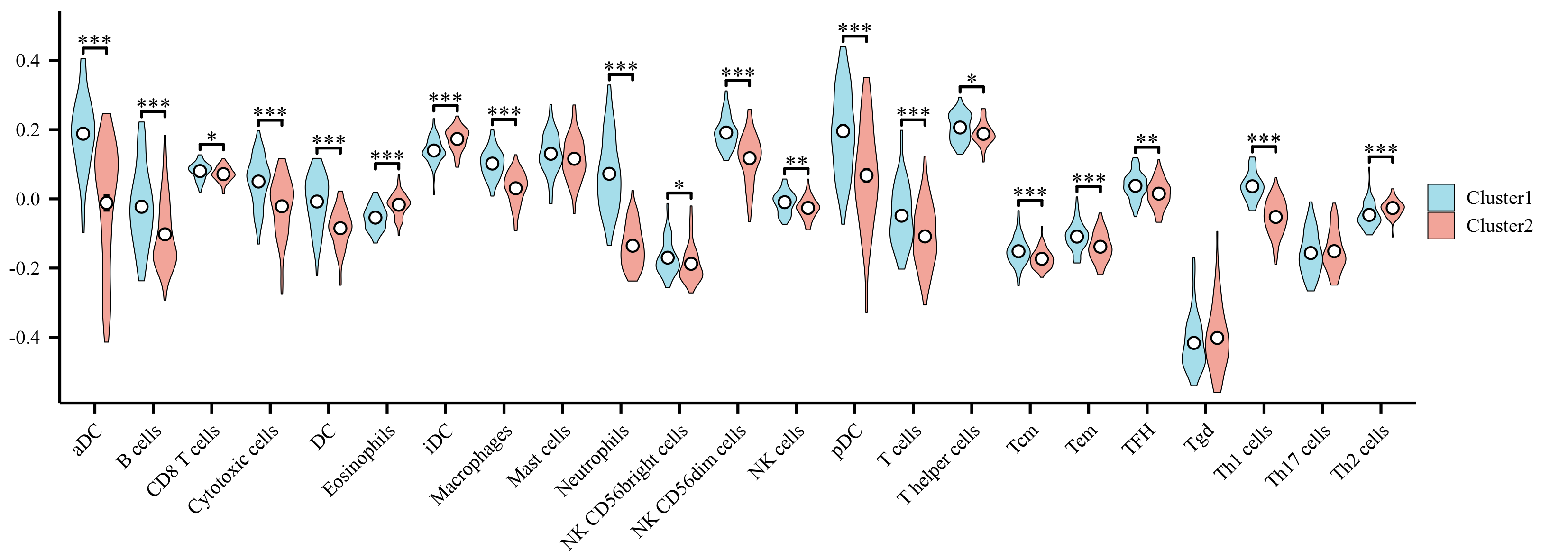

Supplement: S2 Fig — (*P < 0.05, **P < 0.01, ***P < 0.001). (TIF) [file pone.0338880.s002.tif]

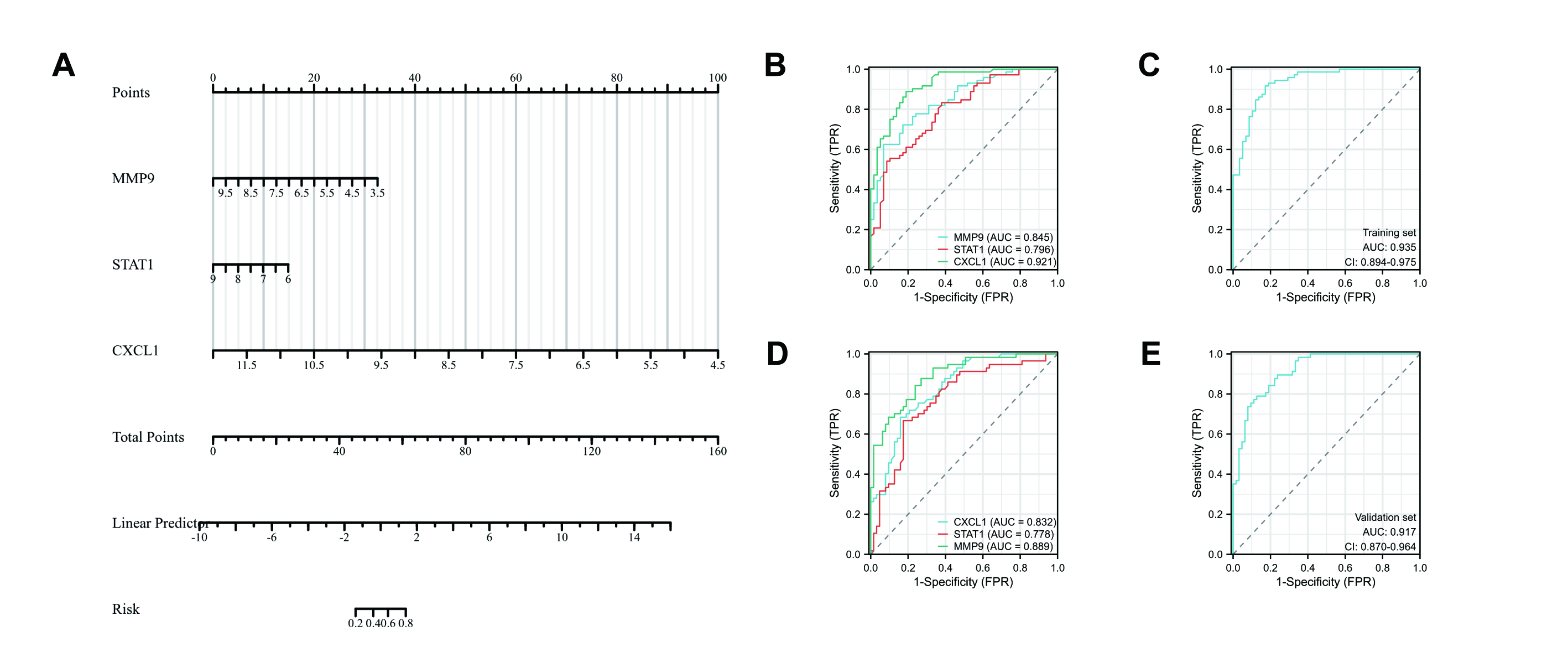

Supplement: S3 Fig — A. Intra-UC group diagnostic scatter plot constructed from the expression levels of three core genes; B. ROC curve for single genes within the UC group in the training set; C. ROC curve for multi-gene combinations in the training set; D. ROC curve for single genes within the UC group in the validation set; E. ROC curve for multi-gene combinations in the validation set. (TIF) [file pone.0338880.s003.tif]

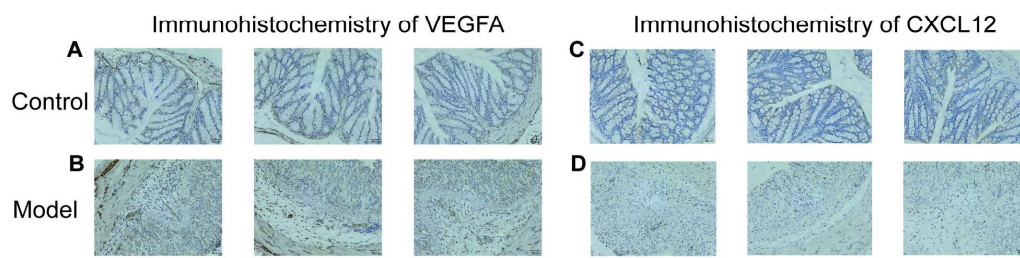

Supplement: S1 Data — (ZIP) [file pone.0338880.s007.zip › Supporting informmation 2 Raw data/Immunohistochemistry.pdf]
