# Supplementary material for: Multi-omics analysis reveals diagnostic and therapeutic biomarkers for aging phenotypes in ulcerative colitis
Source: PLoS One. 2025 Dec 17;20(12):e0338880. doi: 10.1371/journal.pone.0338880 (PMC12711006; doi:10.1371/journal.pone.0338880)
Supplement: S1 Table — (DOCX) [file pone.0338880.s004.docx]

S1 Table. Sample information for the ulcerative colitis transcriptome dataset.

|  | Platforms | Total Sample | UC | Control | Age | Gender | Batch |
| --- | --- | --- | --- | --- | --- | --- | --- |
| GSE38713 | GPL570 | 43 | 22 | 13 | Yes | Yes | No |
| GSE87473 | GPL13158 | 127 | 106 | 21 | Yes | No | No |
| GSE179285 | GPL6480 | 254 | 23 | 23 | No | No | Yes |
| GSE47908 | GPL570 | 60 | 39 | 15 | No | No | No |
| GSE48958 | GPL6244 | 21 | 7 | 8 | No | No | No |
| GSE75214 | GPL6244 | 194 | 74 | 11 | No | No | No |

*All profiling data were derived from colon tissue biopsies. To ensure disease specificity, inactive and non-inflammatory UC samples were excluded from the UC patient group.
