# Supplementary material for: Multi-omics analysis reveals diagnostic and therapeutic biomarkers for aging phenotypes in ulcerative colitis
Source: PLoS One. 2025 Dec 17;20(12):e0338880. doi: 10.1371/journal.pone.0338880 (PMC12711006; doi:10.1371/journal.pone.0338880)
Supplement: S2 Table — (DOCX) [file pone.0338880.s005.docx]

| Gene Name | Primer Sequences（5'-3'） | | Length （bp） |
| --- | --- | --- | --- |
| GADPH | F | CTGGGCTACACTGAGCACC | 148 |
|  | R | AAGTGGTCGTTGAGGGCAATG |  |
| STAT1 | F | ATGCTGGCACCAGAACGAATGAG | 132 |
|  | R | TCACCACAACGGGCAGAGAGG |  |
| CXCL1 | F | TGCTGCTCCTGCTCCTGGTAG | 136 |
|  | R | GGGGACTTCACGTTCACACTTTGG |  |
| MMP9 | F | CCCTGGTCCTGGTGCTCCTG | 113 |
|  | R | CTGCCTGTCGGTGAGATTGGTTC |  |
|  | R | AAGATCCTTGCAGCACCAGTTGG |  |

S2 Table. Primer sequences for quantitative PCR analysis of core genes.
