# Supplementary material for: Multi-omics analysis reveals diagnostic and therapeutic biomarkers for aging phenotypes in ulcerative colitis
Source: PLoS One. 2025 Dec 17;20(12):e0338880. doi: 10.1371/journal.pone.0338880 (PMC12711006; doi:10.1371/journal.pone.0338880)
Supplement: S1 File — (PDF) [file pone.0338880.s006.pdf]

GeneMANIA report

Created on : 29 September 2025 10:02:50
Last database update : 13 August 2021 00:00:00
Application version : 3.6.0

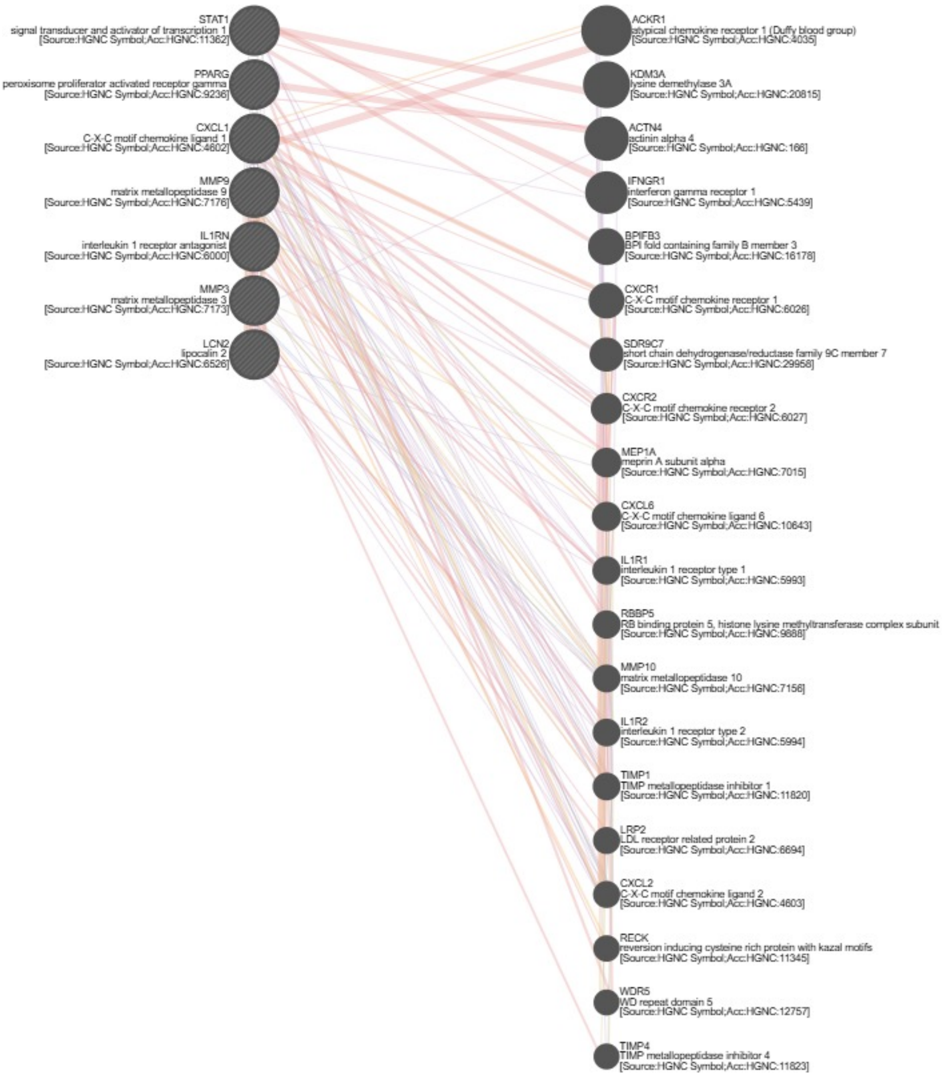

Networks

- Physical Interactions
- Co-expression
- Predicted
- Shared protein domains
- Co-localization

Functions

N/A

Search parameters

Organism Homo sapiens (human)

Genes MMP9 , STAT1 , PPARG , IL1RN , LCN2 , MMP3 , CXCL1

Network weighting Automatically selected weighting method

Networks

A

Abbasi-Schild-Poulter-2019 , Abu-Odeh-Aqeilan-2014 , Achuthankutty-Mailand-2019 , Agrawal-Sedivy-2010 , Ahn-Lee-2008 , Albers-Koegl-2005 , Alexander-Wang-2018 , Alexandru-Deshaies-2008 , Alizadeh-Staudt-2000 , Alsulami-Cagney-2019 , An-Sun-2017 , Andresen-Flores-Morales-2014 , Arbogast-Gros-2019 , Arijs-Rutgeerts-2009 , Arroyo-Aloy-2014 , Arroyo-Aloy-2015 , Asadi-Dhanvantari-2018

B

Bailey-Hieter-2015 , Bandyopadhyay-Ideker-2010 , Banks-Washburn-2016 , Bantscheff-Drewes-2011 , Barr-Knapp-2009 , Barreiro-Alonso-Cerdán-2018 , Barrios-Rodiles-Wrana-2005 , Behrends-Harper-2010 , Behzadnia-Lührmann-2007 , Benleulmi-Chaachoua-Jockers-2016 A , Benleulmi-Chaachoua-Jockers-2016 B , Bennett-Harper-2010 , Benzinger-Hermeking-2005 , Berggård-James-2006 , Bett-Hay-2013 , Beyer-Boldt-2018 , Bhatnagar-Attie-2014 , Bild-Nevins-2006 B , BIOGRID-SMALL-SCALE-STUDIES , BIOGRID-SMALL-SCALE-STUDIES , Bishof-Seyfried-2018 , Blandin-Richard-2013 , Blomen-Brummelkamp-2015 , Blomen-Brummelkamp-2015 , Bogachek-Weigel-2014 , Boldrick-Relman-2002 , Boldt-Roepman-2016 , Botham-Schimmer-2019 , Bouwmeester-Superti-Furga-2004 , Brady-Omary-2018 , Brajenovic-Drewes-2004 , Brehme-Superti-Furga-2009 , Burington-Shaughnessy-2008 , Butland-Hayden-2014 , Byron-Humphries-2012

C

Cai-Conaway-2007 , Camargo-Brandon-2007 , Campos-Reinberg-2015 , Cao-Chinnaiyan-2014 , Carmon-Liu-2014 , Caron-van Attikum-2019 , CELL_MAP , Chen-Brown-2002 , Chen-Ge-2013 A , Chen-Ge-2013 B , Chen-Guan-2018 , Chen-Huang-2014 , Chen-Krogan-2018 , Chen-Yu-2018 , Chen-Zhang-2013 , Chen-Zhou-2019 , Cheng-DeCaprio-2017 , Chi-Reed-2018 , Chitale-Richly-2017 , Choi-Beutler-2019 , Choi-Busino-2018 , Choudhury-Michlewski-2017 , Christianson-Kopito-2011 , Cloutier-Coulombe-2013 , Cloutier-Coulombe-2017 , Colicelli-2010 , Colland-Gauthier-2004 , Conte-Perez-Oliva-2018 , Cooper-Green-2015 , Corominas-Iakoucheva-2014 , Couzens-Gingras-2013 , Cox-Rizzino-2013 , Coyaud-Raught-2015 , Crow-Cristea-2017

D

Daakour-Twizere-2016 , Dabbaghizadeh-Tanguay-2018 , Dart-Wells-2015 , Das-Broemer-2019 , Davis-Glaunsinger-2015 , de Hoog-Mann-2004 , Devarajan-Ketha-Kumar-2012 , Diner-Cristea-2015 , Dittmer-Misteli-2014 , Dobbin-Giordano-2005 ,

D

Douanne-Bidère-2019 , Drissi-Boisvert-2015 , Du-Krogan-2017

E

Elliott-Gyrd-Hansen-2016 , Emdal-Olsen-2015 , Enzo-Dupont-2015 , Ertych-Bastians-2016 , Ewing-Figeys-2007

F

Fang-Lin-2011 , Faust-Frankel-2018 , Fenner-Prehn-2010 , Floyd-Pagliarini-2016 , Foerster-Ritter-2013 , Fogeron-Lange-2013 , Fonseca-Damgaard-2015 , Foster-Marshall-2013 , Fragoza-Yu-2019 , Freibaum-Taylor-2010

G

Gabriel-Baumgrass-2016 , Gallardo-Vara-Bernabeu-2019 , Galligan-Howley-2015 , Gao-Reinberg-2012 , Gao-Vaziri-2016 , Garzia-Sonenberg-2017 , Gautier-Hall-2009 , Giannone-Liu-2010 , Gilmore-Washburn-2016 , Giurato-Tarallo-2018 , Glatter-Gstaiger-2009 , Gloeckner-Ueffing-2007 , Goehler-Wanker-2004 , Gordon-Krogan-2020 , Goudreault-Gingras-2009 , Greco-Cristea-2011 , Grossmann-Stelzl-2015 , Guarani-Harper-2014 , Guard-Old-2019 , Guardia-Laguarta-Przedborski-2019 , Guderian-Grimmler-2011 , Gupta-Pelletier-2015

H

Han-Bassik-2017 A , Han-Bassik-2017 B , Hanson-Clayton-2014 , Hauri-Beisel-2016 , Hauri-Gstaiger-2013 , Havrylov-Redowicz-2009 , Havugimana-Emili-2012 , Hayes-Urbé-2012 , Hegele-Stelzl-2012 A , Hegele-Stelzl-2012 B , Heidelberger-Beli-2018 , Hein-Mann-2015 , Hermjakob-Apweiler-2004 , Herr-Helleday-2015 , Hoffmeister-Längst-2017 , Horlbeck-Gilbert-2018 A , Horlbeck-Gilbert-2018 B , Hosp-Selbach-2015 , Hou-Chen-2018 , Hou-Huang-2017 , Hu-Woods-2019 , Hu-Yin-2019 , Hubel-Pichlmair-2019 , Huber-Hoelz-2017 , HUMANCYC , Humphries-Humphries-2009 , Hussain-Aldaz-2018 , Hutchins-Peters-2010 , Huttlin-Gygi-2015 , Huttlin-Harper-2017 , Hüttenhain-Krogan-2019

I

I2D-BIND-Fly2Human , I2D-BIND-Mouse2Human , I2D-BIND-Rat2Human , I2D-BIND-Worm2Human , I2D-BIND-Yeast2Human , I2D-BioGRID-Fly2Human , I2D-BioGRID-Mouse2Human , I2D-BioGRID-Rat2Human , I2D-BioGRID-Worm2Human , I2D-BioGRID-Yeast2Human , I2D-Chen-Pawson-2009-PiwiScreen-Mouse2Human , I2D-Formstecher-Daviet-2005-Embryo-Fly2Human , I2D-Formstecher-Daviet-2005-Head-Fly2Human , I2D-Giot-Rothbert-2003-High-Fly2Human , I2D-Giot-Rothbert-2003-Low-Fly2Human , I2D-INNATEDB-Mouse2Human , I2D-IntAct-Fly2Human , I2D-IntAct-Mouse2Human , I2D-IntAct-Rat2Human , I2D-IntAct-Worm2Human , I2D-IntAct-Yeast2Human , I2D-Krogan-Greenblatt-2006-Core-Yeast2Human , I2D-Krogan-Greenblatt-2006-NonCore-Yeast2Human , I2D-Li-Vidal-2004-CE-DATA-Worm2Human , I2D-Li-Vidal-2004-CORE-1-Worm2Human , I2D-Li-Vidal-2004-CORE-2-Worm2Human , I2D-Li-Vidal-2004-interolog-Worm2Human , I2D-Li-Vidal-2004-literature-Worm2Human , I2D-Li-

I

Vidal-2004-non-core-Worm2Human , I2D-Manual-Mouse2Human , I2D-Manual-Rat2Human , I2D-MGI-Mouse2Human , I2D-MINT-Fly2Human , I2D-MINT-Mouse2Human , I2D-MINT-Rat2Human , I2D-MINT-Worm2Human , I2D-MINT-Yeast2Human , I2D-MIPS-Yeast2Human , I2D-Ptacek-Snyder-2005-Yeast2Human , I2D-Stanyon-Finley-2004-CellCycle-Fly2Human , I2D-Tarassov-PCA-Yeast2Human , I2D-Tewari-Vidal-2004-TGFb-Worm2Human , I2D-vonMering-Bork-2002-High-Yeast2Human , I2D-vonMering-Bork-2002-Low-Yeast2Human , I2D-vonMering-Bork-2002-Medium-Yeast2Human , I2D-Wang-Orkin-2006-EScmplx-Mouse2Human , I2D-Wang-Orkin-2006-EScmplxIP-Mouse2Human , I2D-Wang-Orkin-2006-EScmplxlow-Mouse2Human , I2D-Yu-Vidal-2008-GoldStd-Yeast2Human , IMID , Ingham-Pawson-2005 , Innocenti-Brown-2011 , INTERPRO , Iradi-Borchelt-2018 , IREF-bhf-ucl , IREF-bind , IREF-bind-translation , IREF-biogrid , IREF-corum , IREF-dip , IREF-hpidb , IREF-hprd , IREF-huri , IREF-innatedb , IREF-intact , IREF-intcomplex , IREF-matrixdb , IREF-mbinfo , IREF-mint , IREF-mppi , IREF-quickgo , IREF-reactome , IREF-SMALL-SCALE-STUDIES , IREF-SMALL-SCALE-STUDIES , IREF-spike , IREF-uniprotpp , IREF-virushost , Ivanochko-Arrowsmith-2019

J

Jain-Parker-2016 , Jang-Trono-2018 , Jeronimo-Coulombe-2007 , Jiang-de Kok-2017 , Jin-Pawson-2004 , Jirawatnotai-Sicinski-2011 , Johnson-Kerner-Wichterle-2015 , Johnson-Shoemaker-2003 , Jones-MacBeath-2006 , Joshi-Cristea-2013 , Jozwik-Carroll-2016 , Jäger-Krogan-2011

K

Kahle-Zoghbi-2011 , Kaltenbach-Hughes-2007 , Kang-Shin-2015 , Karras-Soengas-2019 , Kato-Sternberg-2014 , Katsogiannou-Rocchi-2014 , Kawahara-Paes Leme-2017 , Keller-Lee-2014 , Kennedy-Kolch-2020 A , Kennedy-Kolch-2020 B , Khanna-Parnaik-2018 , Kim-Major-2015 , Kneissl-Grummt-2003 , Koch-Hermeking-2007 , Kotlyar-Jurisica-2015 , Kristensen-Foster-2012 , Kumar-Maddika-2017 , Kumar-Vertegaal-2017 , Kupka-Walczak-2016 , Kärblane-Sarmiento-2015 , Kırlı-Görlich-2015

L

Lambert-Gingras-2015 , Lampert-Peter-2018 , Lau-Ronai-2012 , Lee-Choi-2016 , Lee-Choi-2017 , Lee-Jeong-2017 , Lee-Jou-2019 , Lee-Mayr-2019 , Lee-Songyang-2011 , Lehner-Sanderson-2004 A , Lehner-Sanderson-2004 B , Leung-Jones-2014 , Leung-Miller-2017 , Li-Chen-2015 , Li-Dorf-2011 A , Li-Dorf-2011 B , Li-Dorf-2014 , Li-Fu-2017 , Li-Haura-2013 , Li-Hung-2019 , Li-Lu-2018 , Li-Wang-2016 , Li-Zhou-2017 , Liebelt-Vertegaal-2020 , Lim-Zoghbi-2006 , Lin-Smith-2010 , Lipp-Guthrie-2015 , Liu-Chen-2019 , Liu-Sun-2019 , Liu-Takahashi-2017 , Liu-Tan-2018 , Liu-Varjosalo-2018 , Liu-Wang-2012 , Liu-Xu-2018 , Liu-Yang-2019 , Llères-Lamond-2010 , Loch-Strickler-2012 , Low-Heck-2014 , Lu-Bohr-2017 , Lu-Zhang-2013 , Luck-Calderwood-2020 , Lum-Cristea-2018 , Luo-Elledge-2009

M

Mak-Moffat-2010 , Malinová-Verheggen-2017 , Mallon-McKay-2013 , Malovannaya-Qin-2010 , Malty-Babu-2017 , Markson-Sanderson-2009 , Martin-Elledge-2017 , Maréchal-Zou-2014 , Matsumoto-Nakayama-2005 , Matsuoka-Elledge-2007 , McCracken-Blencowe-2005 , McFarland-Nussbaum-2008 , McNamara-D'Orso-2016 , Meek-Piwnica-Worms-2004 , Menon-Litovchick-2019 , Milev-Mouland-2012 , Miyamoto-Sato-Yanagawa-2010 , Mohammed-Carroll-2013 , Moon-Kim-2014 , Moutaoufik-Babu-2019 , Mugabo-Lim-2018 , Muller-Demeret-2012 , Murakawa-Landthaler-2015

N

Nakamura-Groth-2019 , Nakayama-Ohara-2002 , Napolitano-Meroni-2011 , Narayan-Bennett-2012 , Nassa-Weisz-2019 , Nathan-Goldberg-2013 , NCI_NATURE , Neganova-Lako-2011 , Newman-Keating-2003 , Noguchi-Kawahara-2018 , Nowak-Sommer-2019

O

Oliviero-Cagney-2015 , Oliviero-Cagney-2016 , Olma-Pintard-2009 , Oláh-Ovádi-2011 , Ouyang-Gill-2009

P

Panigrahi-Pati-2012 , Pankow-Yates-2015 , Pao-Virdee-2018 , Papp-Lamia-2015 , Pech-Settleman-2019 , Perez-Hernandez-Yáñez-Mó-2013 , Perez-Perri-Espinosa-2016 , Perou-Botstein-1999 , Perou-Botstein-2000 , Persaud-Rotin-2009 A , Persaud-Rotin-2009 B , Petschnigg-Stagljar-2014 , PFAM , Phillips-Corn-2013 , Pichlmair-Superti-Furga-2011 , Pichlmair-Superti-Furga-2012 , Pilling-Cooper-2017 , Pladevall-Morera-Lopez-Contreras-2019 , Ptushkina-Ray-2017

R

Raisner-Gascoigne-2018 , Ramachandran-LaBaer-2004 , Raman-Harper-2015 , Ramaswamy-Golub-2001 , Ravasi-Hayashizaki-2010 , REACTOME , Reinke-Keating-2010 , Reinke-Keating-2013 , Rengasamy-Walsh-2017 , Reyniers-Taymans-2014 , Richter-Chrzanowska-Lightowlers-2010 , Rieger-Chu-2004 , Rivera-Paes Leme-2018 , Rodriguez-von Kriegsheim-2016 , Roewenstrunk-de la Luna-2019 , Rolland-Vidal-2014 , Rosenbluh-Hahn-2016 , Rosenwald-Staudt-2001 , Ross-Perou-2001 , Roth-Zlotnik-2006 , Rowbotham-Mermoud-2011 , Roy-Pardo-2014 , Roy-Parent-2013 , Rual-Vidal-2005

S

Saez-Vilchez-2018 , Sahni-Vidal-2015 , Saito-Kobarg-2017 , Sala-Ampe-2017 , Salvetti-Greco-2016 , Sang-Jackson-2011 , Sato-Conaway-2004 , Savidis-Brass-2016 , Schadt-Shoemaker-2004 , Schiza-Diamandis-2018 , Scholz-Taylor-2016 , Scifo-Lalowski-2015 , Scott-Guy-2017 , Scott-Schulman-2016 , Shami Shah-Baskin-2019 , Shen-Chen-2019 , Shen-Mali-2017 , Sherman-Teitell-2010 , Simabuco-Zanchin-2019 , Singh-Moore-2012 , So-Colwill-2015 , Sokolina-Stagljar-2017 , Soler-López-Aloy-2011 , Sowa-Harper-2009 , Srivas-Ideker-2016 , St-Denis-Gingras-2015 , St-Denis-

S

Gingras-2016 , Stehling-Lill-2012 , Stehling-Lill-2013 , Stelzl-Wanker-2005 , Stuart-Kim-2003 , Sundell-Ivarsson-2018 , Suter-Wanker-2013 , Swayampakula-Dedhar-2017

T

Taipale-Lindquist-2012 , Taipale-Lindquist-2014 , Takahashi-Conaway-2011 , Tang-Wang-2019 , Tarallo-Weisz-2011 , Teixeira-Gomes-2010 , Teixeira-Laman-2016 A , Teixeira-Laman-2016 B , Thalappilly-Dusetti-2008 , Thompson-Luchansky-2014 , Tiemann-Kani-2019 , Tomkins-Manzoni-2018 , Tong-Moran-2014 , Toyoshima-Grandori-2012 , Trepte-Wanker-2018 A , Trepte-Wanker-2018 B , Tsai-Cristea-2012

U

Ugidos-Vandenbroeck-2019

V

Van Acker-Dewilde-2019 , Van Alstyne-Pellizzoni-2018 , Van Quickelberghe-Gevaert-2018 , van Wijk-Timmers-2009 , Vandamme-Angrand-2011 , Varier-Vermeulen-2016 , Varjosalo-Gstaiger-2013 A , Varjosalo-Gstaiger-2013 B , Varjosalo-Superti-Furga-2013 , Vastrik-Stein-2007 , Venkatesan-Vidal-2009 , Viita-Vartiainen-2019 , Vinayagam-Wanker-2011 , Virok-Fülöp-2011 , Vizeacoumar-Moffat-2013 , von Hundelshausen-Weber-2017

W

Wallach-Kramer-2013 , Wan-Emili-2015 , Wang-Balch-2006 , Wang-Cheung-2015 , Wang-He-2008 , Wang-Huang-2017 , Wang-Liu-2019 , Wang-Maris-2006 , Wang-Xiong-2019 , Wang-Xu-2015 , Wang-Yang-2011 , Watanabe-Fujita-2018 , Weimann-Stelzl-2013 A , Weimann-Stelzl-2013 B , Weinmann-Meister-2009 , Weishäupl-Schmidt-2019 , Weith-Meyer-2018 , Whisenant-Salomon-2015 , Wilkinson-Coba-2019 , Willingham-Muchowski-2003 , Winczura-Jensen-2018 , Wong-O'Bryan-2012 , Woods-Monteiro-2012 A , Woods-Monteiro-2012 B , Woodsmith-Sanderson-2012 , Wu-Garvey-2007 , Wu-Li-2007 , Wu-Ma-2012 , Wu-Stein-2010 , Wu-Stein-2010

X

Xiao-Brown-2018 , Xiao-Lefkowitz-2007 , Xie-Cong-2013 , Xie-Green-2012 , Xie-Zhang-2017 , Xu-Ye-2012 , Xu-Zetter-2016

Y

Yachie-Roth-2016 , Yadav-Varjosalo-2017 , Yamauchi-Maeda-2018 , Yang-Brasier-2015 , Yang-Chen-2010 , Yang-Maurer-2018 , Yang-Vidal-2016 , Yang-Wang-2018 , Yao-Stagljar-2017 A , Yao-Stagljar-2017 B , Yatim-Benkirane-2012 , Yeung-Dougan-2019 , Yu-Chow-2013 , Yu-Engel-2018 , Yu-Vidal-2011 , Yue-Liu-2018

Z

Zanon-Pichler-2013 , Zeller-Wei-2006 , Zhang-Shang-2006 , Zhang-Vermeulen-2017 , Zhang-Wang-2018 , Zhang-Wheeler-2014 , Zhang-Xu-2018 , Zhang-Zou-2011 , Zhao-Krug-2005 , Zhao-Yang-2011 , Zhong-Vidal-2016 , Zhou-Conrads-2004 , Zhou-

Z

Hanemann-2016 , Zhu-Liu-2018

Genes

Gene	Description	Rank
IL1RN	interleukin 1 receptor antagonist [Source:HGNC Symbol;Acc:HGNC:6000]	N/A
LCN2	lipocalin 2 [Source:HGNC Symbol;Acc:HGNC:6526]	N/A
MMP3	matrix metallopeptidase 3 [Source:HGNC Symbol;Acc:HGNC:7173]	N/A
MMP9	matrix metallopeptidase 9 [Source:HGNC Symbol;Acc:HGNC:7176]	N/A
PPARG	peroxisome proliferator activated receptor gamma [Source:HGNC Symbol; Acc:HGNC:9236]	N/A
CXCL1	C-X-C motif chemokine ligand 1 [Source:HGNC Symbol;Acc:HGNC:4602]	N/A
STAT1	signal transducer and activator of transcription 1 [Source:HGNC Symbol; Acc:HGNC:11362]	N/A
ACKR1	atypical chemokine receptor 1 (Duffy blood group) [Source:HGNC Symbol; Acc:HGNC:4035]	1
KDM3A	lysine demethylase 3A [Source:HGNC Symbol;Acc:HGNC:20815]	2
ACTN4	actinin alpha 4 [Source:HGNC Symbol;Acc:HGNC:166]	3
IFNGR1	interferon gamma receptor 1 [Source:HGNC Symbol;Acc:HGNC:5439]	4
BPIFB3	BPI fold containing family B member 3 [Source:HGNC Symbol;Acc:HGNC: 16178]	5
CXCR1	C-X-C motif chemokine receptor 1 [Source:HGNC Symbol;Acc:HGNC:6026]	6
SDR9C7	short chain dehydrogenase/reductase family 9C member 7 [Source:HGNC Symbol;Acc:HGNC:29958]	7
CXCR2	C-X-C motif chemokine receptor 2 [Source:HGNC Symbol;Acc:HGNC:6027]	8
MEP1A	meprin A subunit alpha [Source:HGNC Symbol;Acc:HGNC:7015]	9
CXCL6	C-X-C motif chemokine ligand 6 [Source:HGNC Symbol;Acc:HGNC:10643]	10
IL1R1	interleukin 1 receptor type 1 [Source:HGNC Symbol;Acc:HGNC:5993]	11
RBBP5	RB binding protein 5, histone lysine methyltransferase complex subunit [Source:HGNC Symbol;Acc:HGNC:9888]	12
MMP10	matrix metallopeptidase 10 [Source:HGNC Symbol;Acc:HGNC:7156]	13
IL1R2	interleukin 1 receptor type 2 [Source:HGNC Symbol;Acc:HGNC:5994]	14
TIMP1	TIMP metallopeptidase inhibitor 1 [Source:HGNC Symbol;Acc:HGNC: 11820]	15
LRP2	LDL receptor related protein 2 [Source:HGNC Symbol;Acc:HGNC:6694]	16
CXCL2	C-X-C motif chemokine ligand 2 [Source:HGNC Symbol;Acc:HGNC:4603]	17
RECK	reversion inducing cysteine rich protein with kazal motifs [Source:HGNC Symbol;Acc:HGNC:11345]	18

Gene	Description	Rank
WDR5	WD repeat domain 5 [Source:HGNC Symbol;Acc:HGNC:12757]	19
TIMP4	TIMP metallopeptidase inhibitor 4 [Source:HGNC Symbol;Acc:HGNC: 11823]	20

Networks

Physical Interactions 58.66%

IREF-dip 38.74%

Physical Interactions with 5,037 interactions from iRefIndex

IREF-matrixdb 13.10%

Physical Interactions with 15,422 interactions from iRefIndex

IREF-innatedb 4.53%

Physical Interactions with 2,355 interactions from iRefIndex

IREF-hprd 2.29%

Physical Interactions with 33,375 interactions from iRefIndex

Co-expression 21.73%

Burington-Shaughnessy-2008 4.72%

Tumor cell gene expression changes following short-term in vivo exposure to single agent chemotherapeutics are related to survival in multiple myeloma. Burington et al (2008). *Clin Cancer Res*

Co-expression with 295,320 interactions from GEO

Mallon-McKay-2013 3.10%

StemCellDB: the human pluripotent stem cell database at the National Institutes of Health. Mallon et al (2013). *Stem Cell Res*

Co-expression with 602,113 interactions from GEO

Ramaswamy-Golub-2001 3.06%

Multiclass cancer diagnosis using tumor gene expression signatures. Ramaswamy et al (2001). *Proc Natl Acad Sci U S A*

Co-expression with 284,829 interactions from supplementary material

Arijs-Rutgeerts-2009 1.94%

Mucosal gene expression of antimicrobial peptides in inflammatory bowel disease before and after first infliximab treatment. Arijs et al (2009). *PLoS One*

Co-expression with 676,695 interactions from GEO

Wang-Cheung-2015 1.80%

Genetic variation in insulin-induced kinase signaling. Wang et al (2015). *Mol Syst Biol*

Co-expression with 422,896 interactions from GEO

Bild-Nevins-2006 B 1.68%

Oncogenic pathway signatures in human cancers as a guide to targeted therapies. Bild et al (2006). *Nature*

Co-expression with 285,368 interactions from GEO

Boldrick-Relman-2002 1.19%

Stereotyped and specific gene expression programs in human innate immune responses to bacteria. Boldrick et al (2002). *Proc Natl Acad Sci U S A*

Co-expression with 116,197 interactions from supplementary material

Rosenwald-Staudt-2001 1.08%

Relation of gene expression phenotype to immunoglobulin mutation genotype in B cell chronic lymphocytic leukemia. Rosenwald et al (2001). *J Exp Med*

Co-expression with 118,097 interactions from supplementary material

Co-expression 21.73%

Alizadeh-Staudt-2000 1.05%

Distinct types of diffuse large B-cell lymphoma identified by gene expression profiling. Alizadeh et al (2000). *Nature*

Co-expression with 92,360 interactions from supplementary material

Wu-Garvey-2007 0.97%

The effect of insulin on expression of genes and biochemical pathways in human skeletal muscle. Wu et al (2007). *Endocrine*

Co-expression with 275,155 interactions from GEO

Dobbin-Giordano-2005 0.93%

Interlaboratory comparability study of cancer gene expression analysis using oligonucleotide microarrays. Dobbin et al (2005). *Clin Cancer Res*

Co-expression with 452,322 interactions from GEO

Jiang-de Kok-2017 0.21%

Omics-based identification of the combined effects of idiosyncratic drugs and inflammatory cytokines on the development of drug-induced liver injury. Jiang et al (2017). *Toxicol Appl Pharmacol*

Co-expression with 444,959 interactions from GEO

Predicted 11.91%

Wu-Stein-2010 11.91%

A human functional protein interaction network and its application to cancer data analysis. Wu et al (2010). *Genome Biol*

Predicted with 89,967 interactions from supplementary material

Shared protein domains 6.26%

INTERPRO 3.66%

Shared protein domains with 621,159 interactions from InterPro

PFAM 2.60%

Shared protein domains with 471,533 interactions from Pfam

Co-localization 1.44%

Johnson-Shoemaker-2003 1.44%

Genome-wide survey of human alternative pre-mRNA splicing with exon junction microarrays. Johnson et al (2003). *Science*

Co-localization with 426,464 interactions from GEO
