# Supplementary material for: Multi-omics analysis reveals diagnostic and therapeutic biomarkers for aging phenotypes in ulcerative colitis
Source: PLoS One. 2025 Dec 17;20(12):e0338880. doi: 10.1371/journal.pone.0338880 (PMC12711006; doi:10.1371/journal.pone.0338880)
Supplement: S1 Data — (ZIP) [file pone.0338880.s007.zip › Supporting informmation 2 Raw data/WB.pdf]

CXCL12

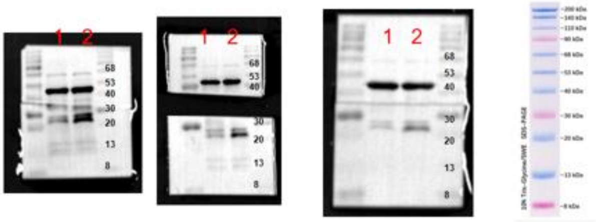

CXCL12
28kDa
Wableibio
(shenyang, China)
WL02283

MMP9

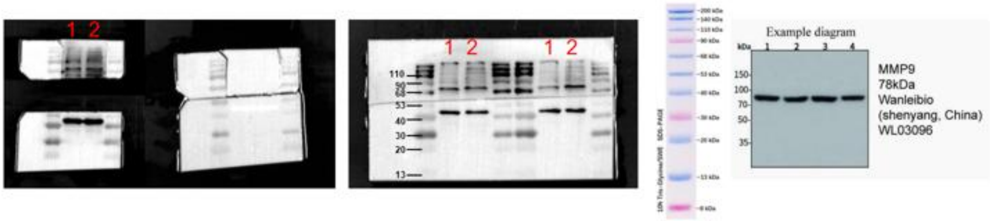

STAT1

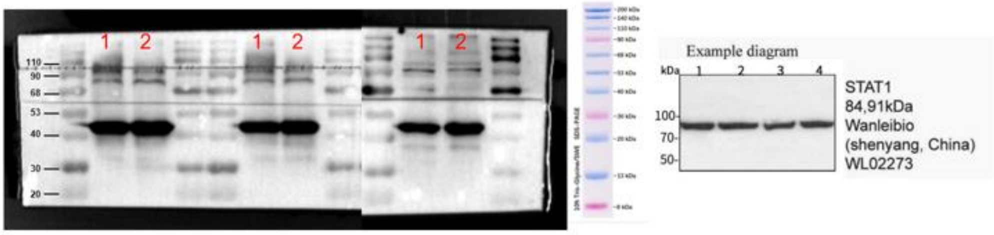

P-STAT1

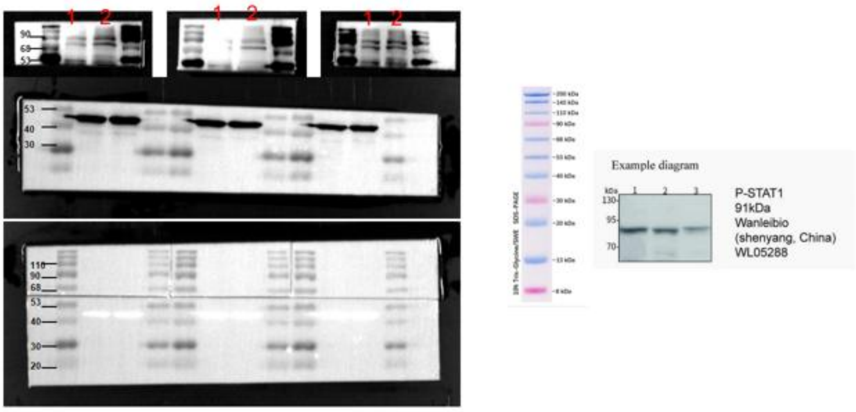

VEGFA

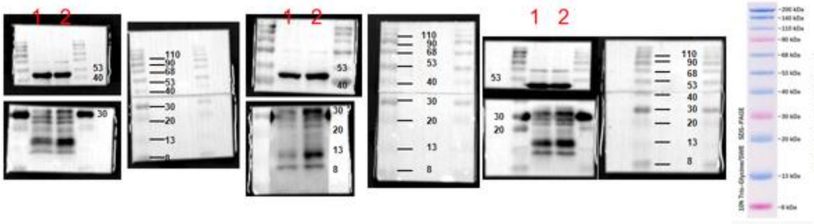

VEGFA
27kDa
HUABIO
(hangzhou, China)
ET1604-28

CXCL12

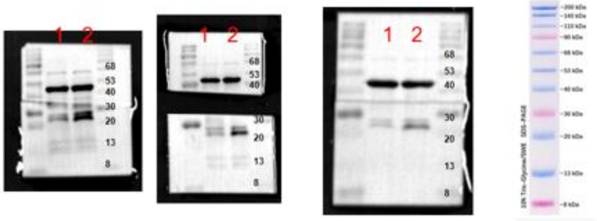

CXCL12
28kDa
Wableibio
(shenyang, China)
WL02283
